# Personal Growth and Life Satisfaction during Fertility Treatment—A Comparison between Arab and Jewish Women

**DOI:** 10.3390/ijerph20032187

**Published:** 2023-01-25

**Authors:** Salam Abu-Sharkia, Orit Taubman - Ben-Ari, Ali Mofareh

**Affiliations:** 1 The Louis and Gabi Weisfeld School of Social Work, Bar Ilan University, Ramat Gan 5290002, Israel; 2 Clalit Health Services (Kupat Holim Clalit), Jerusalem 9112102, Israel

**Keywords:** infertility, life satisfaction, perceived stigma, personal growth

## Abstract

Coping with difficulty conceiving and the ensuing fertility treatments is a stressful experience that impacts many aspects of women’s lives. On the basis of Lazarus and Folkman’s model of stress and coping and Schaefer and Moos’s model of personal growth, and in view of the sparse literature on cultural aspects of infertility and personal growth, this study examined the relationship between stress on the one hand and personal growth and life satisfaction on the other among Arab and Jewish Israeli women. Furthermore, it investigated the moderating role played by perceived stigma, coping flexibility, cultural orientation (individualism and collectivism), and ethnicity. Two hundred five Arab and Jewish Israeli women undergoing fertility treatment completed self-report questionnaires. The results show that Arab women reported higher levels of personal growth and individualism than Jewish women. In the whole sample, a linear negative relationship was found between stress and life satisfaction, and a curvilinear relationship was found between stress and personal growth. In addition, perceived stigma, collectivism, individualism, and coping flexibility were found to moderate the association between perceived stress and personal growth. The findings provide further understanding of personal growth in the context of infertility, showing that personal resources and perceptions are more important than cultural differences in this regard.

## 1. Introduction

Coping with difficulty conceiving and the ensuing fertility treatments is a highly stressful experience that impacts various aspects of a woman’s life. Even after successful treatment, self-image, familial and social relationships, and perceptions of life may be adversely affected [1,2]. According to Lazarus and Folkman’s [3] model of stress and coping, when individuals experience stress, they make consistent cognitive and behavioral efforts to restore balance in light of the new external and/or internal demands on their resources, whether physical, social, or psychological. In other words, successful coping depends on the resources available to them and is reflected in their satisfaction with life. Furthermore, in the past twenty years, research has indicated the potential positive changes that may occur in the wake of coping with a significant life crisis, namely personal growth [4,5]. Recent research has shown that fertility treatments may also lead to positive psychological outcomes, representing an opportunity for empowerment and personal growth for both the individual and the couple [6,7]. However, previous studies have paid scant attention to cultural aspects in this context.

The current study draws on both Lazarus and Folkman’s [3] model of stress and coping and Schaefer and Moos’s [4] model of personal growth in an attempt to identify personal and cultural characteristics (coping flexibility, cultural orientation, stigma, and ethnicity) that may moderate the association between perceived stress on the one hand and personal growth and life satisfaction on the other among women undergoing fertility treatment. The sample consisted of Jewish and Arab women in Israel, enabling us to compare women from different cultures reflected by their ethnicities.

### 1.1. Infertility and Stress

The inability to conceive or failure to carry a baby to term after at least one year of regular and unprotected sexual intercourse is challenging and stressful in most societies [8]. Israel is considered a leader in in vitro fertilization (IVF) and ranks first in the world in the use of this technology [9]. Indeed, assisted reproductive technologies are supported by the state, so that every Israeli woman aged 18 to 45 is entitled to unlimited, subsidized treatments up to the birth of two live children with her current partner, if applicable, irrespective of her family status or sexual orientation [10]. It is currently estimated that 15% of couples in their twenties in developed countries are diagnosed with fertility difficulties [11], and the same rate is reported in Israel.

Couples coping with fertility problems often report emotional distress [12]. The sense of failure, uncertainty, and lack of control over the treatment, along with the impact on the couple’s plans, the long wait for results, and the financial costs, place a heavy burden on their resources and can harm the intimacy of their relationship [13,14,15]. Moreover, research indicates that couples feel threatened by the medical terminology and the technical aspects of fertility treatment, particularly when there is a language barrier, such as when they belong to a cultural minority [16]. Some couples describe a withdrawal from social interactions due to the fear of revealing the problem to their friends and family, coupled with feelings of envy and rivalry. This alienation from their social group may prevent them from receiving support when they need it most [17], thus increasing their stress. Nevertheless, researchers have begun to recognize that fertility treatment may also lead to positive psychological outcomes, including personal growth and enhanced life satisfaction [18,19].

### 1.2. Personal Growth and Life Satisfaction

Personal growth [4], also called posttraumatic growth [20], is defined as positive and meaningful psychological changes which take place subsequent to a stress-related life event and occur as a result of coping with the experience. Growth can manifest itself in one or more of five ways: a closer bond with others; recognizing new life possibilities; a stronger sense of personal strength; increased spirituality; and a renewed appreciation of life [20].

Recent studies reveal the complex curvilinear relationship between perceived stress and growth. They show that intermediate levels of stress are related to higher levels of personal growth [21]. Rozen and colleagues [21] suggest that a low level of stress is insufficient to stimulate growth, while a high level of stress demands a large investment of mental and cognitive resources in order to regulate and process emotions, thereby reducing the degree of emotional availability required for the emergence of growth.

In recent years, researchers have begun to examine personal growth following a variety of stressful, although nontraumatic, life events, including fertility problems [7]. Although research in this field is still in its initial stages, there is evidence that women may experience personal growth in the wake of fertility treatment, in the form of changed self-perception and improved relationships [22].

Life satisfaction reflects the way in which the individual perceives and values their life [23] with respect to a range of domains, including family, interpersonal relationships, work, and studies [24]. Difficulty in any one of these aspects can undermine the sense of life satisfaction in general. Studies suggest that fertility treatment is detrimental to life satisfaction [25], with women undergoing treatment reporting lower satisfaction in life [26].

Although research indicates links between fertility treatment and both personal growth and life satisfaction, the factors that contribute to these positive outcomes are still unclear. Tedeschi and Calhoun [20] contend that individual characteristics, social support, and cognitive processing are important variables closely associated with positive changes. According to Schaefer and Moos [4], four sets of factors contribute to growth: personal characteristics, characteristics of the environment, characteristics of the life event, and coping responses. In the current study, we focused on coping flexibility, which not only represents a personal characteristic, but also relates to the cognitive processing of coping strategies.

### 1.3. Coping Flexibility

Studies demonstrate that the ability to be flexible, that is, to utilize different strategies as specific situations require, constitutes a resilience factor for coping with stress-related events [27]. It has been found, for example, that among bereaved siblings, a higher level of coping flexibility was associated with less distress and more posttraumatic growth [28].

Studies conducted among women undergoing fertility treatment show that those who act in a more flexible way by employing several strategies of active coping report less stress and more positive (re)thinking [13]. Furthermore, women who set new goals and pursued new activities as a means of adaptation displayed lower levels of anxiety and depression than those who focused exclusively on the attempts to conceive [29]. This was true even after 3–5 years of unsuccessful treatments. These reports suggest that a higher level of coping flexibility might be associated with both personal growth and greater satisfaction with life. However, personal resources cannot explain the whole picture; cultural characteristics should also be considered.

### 1.4. Infertility and Cultural Orientation: Individualism vs. Collectivism

The repercussions of fertility problems frequently result from social constructions rather than from the medical problem itself [30], indicating the important role of the sociocultural context in shaping the experience of coping with infertility. Israel is a familial society that highly values parenthood. The wide availability and prevalence of fertility treatments are signs of the social pressure promoting procreation [10] in both Jewish and Arab societies. However, there are cultural differences between the two populations.

The biblical commandment “be fruitful and multiply” is central to Judaism. Consequently, considerable medical and financial efforts are invested in bringing children into the world, both on the national and the individual level. Moreover, similar to the situation in many Western countries, Jewish Israeli society tends toward individualism, with the family generally characterized by democratic relationships and relatively permissive parental control. This reflects the belief that life experience, hardship, and adversities shape one’s personality, an attitude that encourages tackling challenges, confronting problems, and choosing an active and direct way of coping [10,31].

On the other hand, the Arab population in Israel (Muslims, Christians, and Druze), is in transition from a traditional to a more modern society. Despite the process of modernization, the family and collective are still favored over the individual [32]. This tendency to collectivism means that people see themselves and their life goals as integral to their family and community, and therefore involve themselves in social life and tend to rely on it at times of hardship [33]. Arab families prioritize families’ over individuals’ needs; they believe in predestiny and tend to regard illness, including infertility, as caused by metaphysical forces [31].

Among Muslim couples, a woman’s infertility can serve as legitimate grounds for divorce, or for the taking of a second wife by the husband, which is permitted by Islamic law [34]. When it is the man who is infertile, many couples avoid sharing this information, and their wives may take the “blame” and bear the stigma before the family and society [34,35].

### 1.5. Stigma

A stigma is a negative perception that differentiates a person from others. It includes three interdependent components: cognitive (the stereotype); emotional (prejudice); and behavioral (discrimination). Negative beliefs and feelings toward an individual or group may lead to discriminatory behavior, which, in turn, preserves and inflames existing stigmas and prejudices [36]. Research has shown that 49% of those who turn to fertility clinics are concerned about the associated stigma [37]. In a family-oriented society such as Israel, fertility problems per se constitute a stigma and may lead to varying levels of emotional distress. Many couples choose not to confide even in family members or close friends [34,38], thereby preventing the receipt of support.

This problem may be even more severe in Arab society, where parenthood is generally expected to follow immediately on marriage and motherhood is viewed as the most meaningful experience in a woman’s life [39]. Thus, Arab women dealing with fertility problems are less likely than Jewish women to receive support from their families, a factor found to encourage personal growth [40] and decrease life satisfaction [41].

### 1.6. The Current Study

This study examined the associations between stress and personal growth and life satisfaction among Israeli women undergoing fertility treatment, comparing Jewish and Arab women and exploring the moderating role of several personal and cultural factors. In light of the literature, we hypothesized the following:

(1) A negative linear relationship would be found between level of stress and life satisfaction, so that the higher the level of stress, the lower the satisfaction with life.
(2) A curvilinear relationship would be found between level of stress and personal growth, so that moderate levels of stress would be associated with higher levels of growth and high or low levels of stress would be associated with lower levels of growth.
(3) Coping flexibility would be positively associated with both life satisfaction and personal growth, so that the higher the level of coping flexibility, the higher the satisfaction with life and growth.

In addition, given the lack of previous findings on which to rely, the following questions were investigated exploratively:

(1) Is there a difference in the levels of stress, personal growth, and life satisfaction between Arab and Jewish women?
(2) Is the cultural orientation associated with personal growth and life satisfaction?
(3) Is the perceived stigma attached to fertility treatment associated with personal growth and life satisfaction?
(4) What is the unique and combined contribution of the study variables to personal growth and life satisfaction?
(5) Do coping flexibility, cultural orientation (collectivism/individualism), perceived stigma, and ethnicity (Arab, Jewish) moderate the association between stress on the one hand and personal growth and life satisfaction on the other?

## 2. Method

### 2.1. Participants

The sample consisted of 205 women aged 18–40 from throughout Israel who were undergoing fertility treatment. Of these, 105 were Arab and 100 were Jewish. Most were married and did not have children. The distribution of the participants in both groups on the categorical and continuous sociodemographic variables is presented in Table 1 and Table 2. As the tables indicate, economic status, years of education, years of marriage, and number of treatment cycles were similar in the two study groups. However, Arab women and their spouses were younger and had been in treatment for a longer time, and a higher proportion did not yet have children and claimed that the problem lay with their partner. On the other hand, more Jewish women were secular and were employed outside the home.

### 2.2. Instruments

**Perceived Stress Scale** (PSS) [42] includes 14 items concerning the participant’s feelings and thoughts during the last month (e.g., “How often have you felt nervous and ‘stressed’?”). Replies are indicated on a 4-point scale ranging from 1 (never) to 4 (very often). The Cronbach’s alpha was 0.88 in a previous study [21] and 0.84 in the current study. Thus, after reverse-coding the positively phrased items, a total score was calculated by averaging the responses to all items, with higher scores indicating a higher level of stress.

**Posttraumatic Growth Inventory** (PTGI) [43] was used to measure personal growth. The 21 items relate to changes concerning new possibilities, relating to others, personal strength, spiritual change, and appreciation of life. The participants were asked to indicate the degree to which they had experienced the change described in each item since beginning fertility treatment, marking their responses on a 6-point Likert scale ranging from 0 (not at all) to 5 (to a very great degree). Cronbach’s alpha of 0.90 was reported by the scale’s developers, and the same was found in the current study. A score was calculated for each participant by averaging her responses to all items, with higher scores indicating greater personal growth.

**Satisfaction with Life Scale** (SWLS) [44] consists of 5 items (e.g., “In most ways my life is close to my ideal”) marked on a 7-point Likert scale from 1 (strongly disagree) to 7 (strongly agree). The Cronbach’s alpha of the original scale was 0.87 (Diener et al., 1985), and that of the current study was 0.83. A total score was calculated by averaging the responses to all items, with higher scores indicating a higher level of life satisfaction.

**Coping Flexibility Questionnaire** (COFLEX) [45] contains 13 items assessing the ability to adapt and demonstrate versatility and reflective coping. The participants were asked to indicate how often they respond to serious problems in the manner described in each item, using a 4-point Likert scale from 1 (rarely or never) to 4 (almost always). Cronbach’s alpha of 0.78 was reported for the original scale, and the same was found in the current study. A score was calculated for each participant by averaging her responses to all items, with higher scores indicating greater coping flexibility.

**Individualism–Collectivism Scale** (ICS) [46], used to examine the participant’s cultural orientation, contains 32 items reflecting two cultural models: individualism (16 items; e.g., “I’d rather depend on myself than others”) and collectivism (16 items; e.g., “Parents and children must stay together as much as possible”). Responses are indicated on a 5-point scale ranging from 1 (strongly disagree) to 5 (strongly agree). The authors of the questionnaire report Cronbach’s alphas of 0.81 for individualism and 0.76 for collectivism [46]. In the current study, they were 0.67 and 0.63, for individualism and collectivism, respectively. Each of the participants was assigned a score for each measure equal to the mean of her responses to the relevant items, with higher scores indicating a higher level of individualism or collectivism.

**Stigma Scale for Receiving Psychological Help Questionnaire** (SSRPH) [47], adapted for women contending with infertility [48], consists of 5 items (e.g., “Receiving treatments for fertility problems carries a social stigma”), with a 4-point response scale ranging from 0 (do not agree) to 3 (very much agree). The Cronbach’s alpha was 0.76 in a previous study [48] and 0.67 in the current study. A score was calculated for each participant by averaging her responses to all items, with higher scores indicating higher perceived infertility-related stigma.

**A sociodemographic questionnaire** was used to obtain data regarding the participants’ personal and familial characteristics, including age, spouse’s age, education, years of marriage, religion, level of religiosity, period of infertility, cause of infertility, and stage of treatment.

### 2.3. Procedure

After receiving the approval of the University Review Board, the data were collected by posting a request in Hebrew and Arabic on social media and Internet parenting forums along with a link to the questionnaire. The criteria for inclusion in the study were being a woman of 18 or older with fertility problems who was currently undergoing treatment. The women were assured that participation was voluntary, that they could stop completing the questionnaire whenever they wished, and that no identifying information would be collected. Due to the method of data collection, the response rate cannot be assessed. A total of 225 questionnaires were received. After exclusion of those that were not fully completed or were submitted by women who did not meet the study criteria, 205 questionnaires were included in the analysis.

### 2.4. Data Analysis

All analyses were conducted using IBM SPSS version 27. First, one-way ANOVAs were conducted to compare the research groups (Research Question 1). Next, Pearson correlations were calculated to examine the relationships between the variables (Hypotheses 1 and 3 and Research Questions 2 and 3). Then, the unique and combined contribution of the study variables to personal growth and life satisfaction (Research Question 4) and the moderating role played by coping flexibility, cultural orientation, and stigma (Research Question 5) were examined by means of two hierarchical regressions. Where significant interactions were found, their source was examined by PROCESS, a procedure for examining direct and indirect effects [49]. In both regressions, the variables were entered in forced order. In Step 1, study group (Arab or Jewish) was entered together with the background characteristics found to correlate significantly with the research variables (age, economic status, months since start of treatment, number of treatment cycles, having children or not). Despite the strong connection between the number of treatment cycles and the time since the start of treatment (r = 0.57, *p* < 0.001), a multicollinearity test indicated that no problem was created by using both of these independent variables. Perceived stress was entered in Step 2, and coping flexibility, cultural orientation, and perceived stigma were entered in Step 3. In Step 4, moderation was examined by five interactions: stress X perceived stigma; stress X coping flexibility; stress X individualism; stress X collectivism; and stress X ethnicity. Finally, the possibility of a curvilinear relationship between stress and personal growth (Hypothesis 2) was examined by means of a 3-step hierarchical regression, with background variables entered in Step 1, a linear relationship between stress and growth in Step 2, and a quadratic relationship between these variables in Step 3.

In a preliminary stage, the missing data were completed by a process of multiple imputation. To ensure that the data completion remained unbiased, we examined the missing values template. Dividing the sample between the two populations showed that values were missing completely at random [50] in both groups. Hence, the completion process was conducted for each group separately. It should be noted that the missing value rate was not high (less than five percent), the only exception being the number of fertility cycles, which was found missing for over ten percent of the women. Little Test for Missing Completely at Random (MCAR) [51] indicated full randomness both for the Jewish and Arab participants combined and for each group individually (Arab: chi-square = 145.46, df = 122, *p* = 0.073; Jewish: chi-square = 104.41, df = 86, *p* = 0.086).

## 3. Results

### 3.1. Differences between Study Groups

The means and standard deviations of the variables in the two study groups, along with the results of the ANOVAs comparing the groups (Research Question 1), are presented in Table 3. As can be seen from the table, personal growth and individualism were higher among Arab women than among Jewish women. In addition, Arab women reported greater coping flexibility and perceived a greater stigma attached to fertility treatment. No other differences were found between the groups.

### 3.2. Associations between Study Variables

The results of the Pearson correlations between the study variables for each study group for the whole sample appear in Table 4. As the table shows, a higher level of stress was significantly related to lower life satisfaction in both groups, as predicted in Hypothesis 1. Moreover, in confirmation of Hypothesis 3, among all participants, greater coping flexibility was significantly associated with higher personal growth and with higher life satisfaction. In addition, a higher level of collectivism was significantly associated with both personal growth and life satisfaction (Research Question 2). No relationship was found between perceived stigma and either personal growth or life satisfaction (Research Question 3).

### 3.3. Contribution of the Study Variables to Personal Growth and Life Satisfaction

Two four-step hierarchical regressions were conducted to examine the unique and combined contribution of the independent variables to personal growth and life satisfaction (Research Questions 4 and 5). The results are presented in Table 5.

The regression for personal growth showed that the variables explained 40.3% of the variance in this outcome. In Step 1, the background variables contributed 15% to the explained variance, with being an Arab and higher economic status both associated with greater growth. Stress in Step 2 contributed a further 5.4% to the explanation of the variance, so that the higher the level of stress, the lower the level of personal growth. In Step 3, coping flexibility, stigma, and cultural orientation (collectivism/individualism) added 11.9% to the explained variance, with higher coping flexibility and collectivism significantly associated with higher personal growth. In Step 4, with an additional 8% of explained variance, four interactions between stress on the one hand and stigma, coping flexibility, individualism, and collectivism on the other were found to be significant. The interaction between stress and ethnicity was not significant.

**Figure 1:**
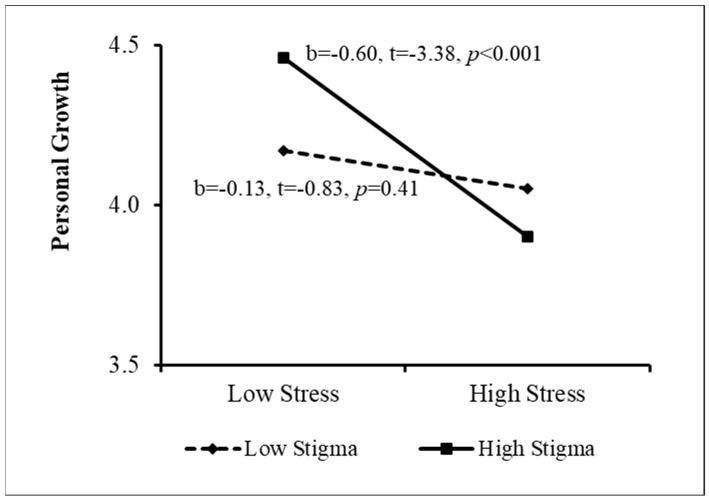
Sources of the Interaction between Stress and Perceived Stigma.

**Figure 2:**
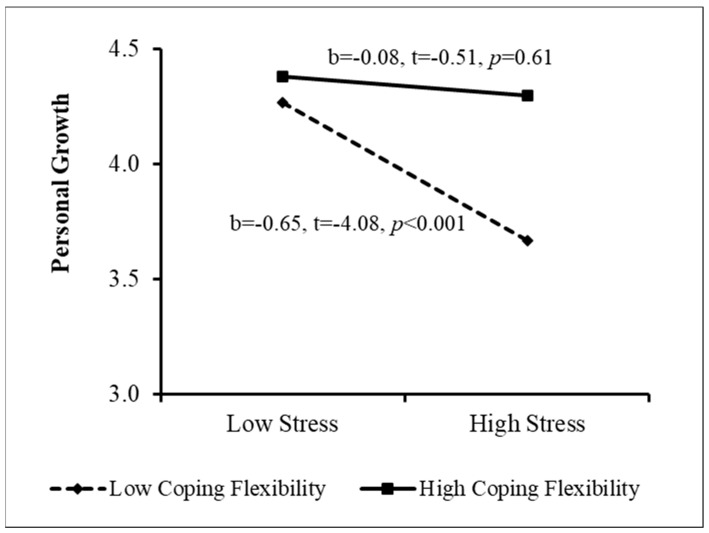
Sources of the Interaction between Stress and Coping Flexibility.

**Figure 3:**
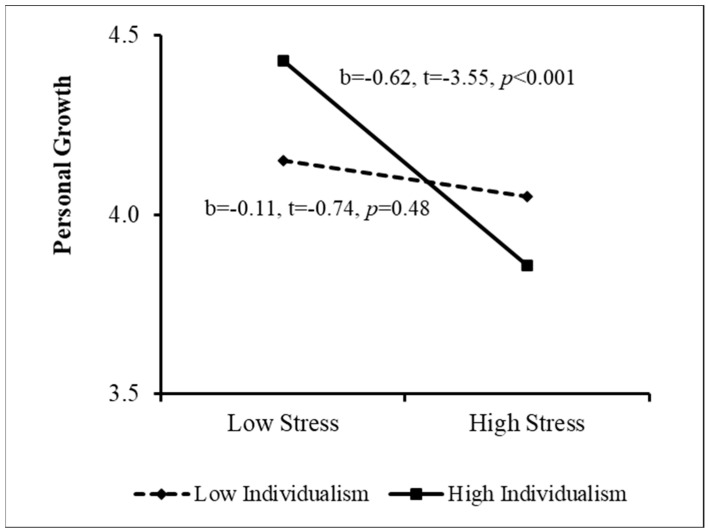
Sources of the Interaction between Stress and Individualism.

**Figure 4:**
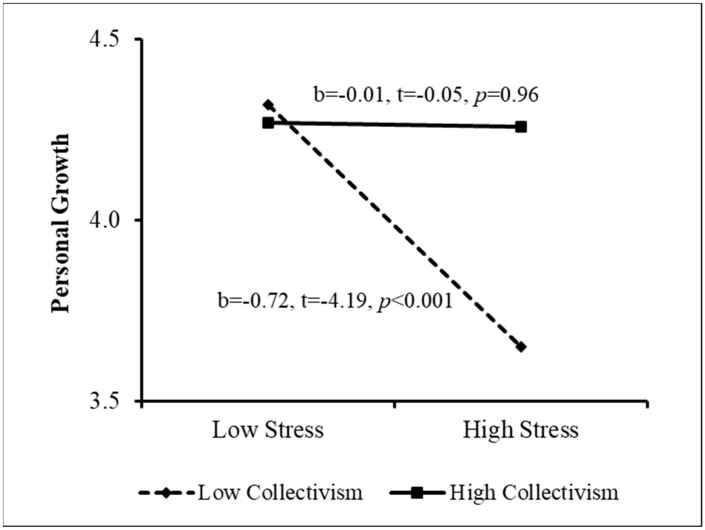
Sources of the Interaction between Stress and Collectivism.

The PROCESS procedure [49] was then used to examine the sources of the interactions. The results appear in Figure 1, Figure 2, Figure 3 and Figure 4. As Figure 1 indicates, when perceived stigma was high, the association between stress and personal growth was negative and stronger than that when perceived stigma was low. In other words, among women who sensed greater stigmatization because of their need for fertility treatment, greater stress was associated with lower growth. This effect was much weaker among women reporting low perceived stigma. In addition, when coping flexibility was low, the relationship between stress and personal growth was negative, but it was insignificant when coping flexibility was high (Figure 2). That is, among women displaying lower coping flexibility, greater stress was related to lower personal growth. However, no relationship was found between stress and growth among women demonstrating higher coping flexibility. Figure 3 shows that among women reporting higher levels of individualism, the association between stress and personal growth was negative, while this association was insignificant among women reporting low individualism. Finally, when the level of collectivism was low, a negative relationship was found between stress and personal growth, and it was insignificant among women reporting high collectivism (see Figure 4). That is, only among women reporting a lower level of collectivism, the greater the stress, the lower the level of growth (Research Question 4). In step 5, the moderation effect of ethnicity was not found to be significant.

With respect to life satisfaction, the independent variables explained 47.2% of the variance. In Step 1, the background variables accounted for 21.7% of the variance, showing that the better the woman’s economic status and the fewer treatment cycles she had undergone, the higher her life satisfaction. Stress in Step 2 contributed an additional 18.8% to the explained variance, with higher stress associated with lower life satisfaction. Step 3 added 5.6% to the explanation of the variance: higher coping flexibility and collectivism were associated with higher life satisfaction. None of the interactions entered in Step 4 were found to make a significant contribution to explaining the variance in life satisfaction (Research Question 5).

### 3.4. A Curvilinear Association between Stress and Personal Growth

**Figure 5:**
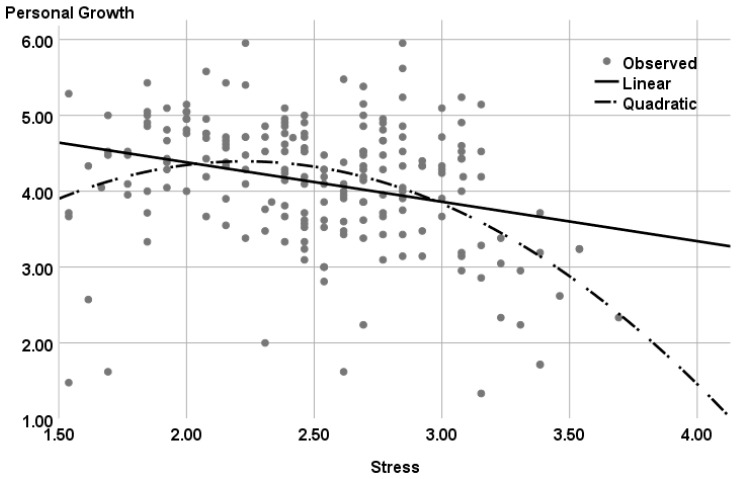
Curvilinear Relationship between Stress and Personal Growth.

A further hierarchical regression was performed to examine Hypothesis 2 concerning the association between stress and personal growth. The results of this analysis appear in Table 6 and Figure 5.

As Table 6 indicates, the curvilinear relationship between stress and personal growth entered in Step 3 contributed a significant 7.2% to the explained variance. In other words, women who experienced a moderate level of stress reported the greatest growth. In contrast, the more extreme the level of stress (either very high or very low), the less growth was reported.

## 4. Discussion

The current study sought to examine the implications of the stress evoked by fertility treatments on the personal growth and life satisfaction of Jewish and Arab women and investigate the moderating role played by perceived stigma, coping flexibility, cultural orientation, and ethnicity. Although the psychological consequences of infertility have been discussed extensively [8,14,15], cultural aspects have yet to receive sufficient attention, especially with respect to personal growth. The findings of the current study therefore constitute an important addition to the growing body of knowledge on the effects of fertility treatments.

### 4.1. Comparison between Arab and Jewish Women

The findings indicate a greater inclination toward individualism, perceived stigma, coping flexibility, and personal growth among Arab women in fertility treatment than among Jewish women. The fact that Arab women perceived themselves to be more individualistic than did the Jewish participants is surprising given that Jewish society as a whole is considered more individualistic than Arab society, which is typically described as collectivist [52,53]. However, it supports Joseph’s [54] claim that under certain circumstances, and despite familial group norms, Arab individuals and families have cultivated dynamic ideas about the self. Therefore, despite the dominant tendency to be invested in the collective, the “I” is also an active agent and not merely the passive product of the values, concepts, and constructs of family kinship.

Furthermore, difficulty conceiving may undermine an Arab woman’s sense that her life is clearly mapped out, dictated by the norms of procreation and family expansion, which are seen not only as supreme values [55], but also as a social obligation [56]. In addition, Arab women tend not to disclose their fertility problems, even to their closest friends and family members [34,38], which might distance them from their social network, thereby diminishing the power of these relationships and creating isolation. This distance may be interpreted as a need to take greater control of their lives and demonstrate independence, reflected in a higher sense of individualism, and maybe also encouraging them to endorse greater coping flexibility. Hence, in this context, Arab women’s tendency toward individualism, which usually reflects the belief that life experience, including adversities, shapes one’s personality, encouraging a choice of an active and direct way of coping, may be considered here as a coping mechanism for dealing with a highly judgmental society, what Dwairy [57] calls a type of self-imposed “emotional exile”, meaning that they tend to be more dependent on themselves than on the society, and adopted in order to cope with the stigmatization and social pressures they experience.

This explanation is supported by the finding that Arab participants reported higher perceived stigma than Jewish women. As the demand for procreation is anchored in religion and tradition, any deviation from the norm is negatively perceived [39]. This finding is in line with findings from Kuwait, indicating that women contending with infertility experience aggressive accusations, stigma, mental distress, and social ostracism [58].

Within this complex social reality, the Arab participants in our study also reported greater growth than the Jewish participants, most of whom defined themselves as secular. Research shows that the resources of religion and religious belief moderate the negative influences of stressful life events in general and coping with fertility problems in particular [59,60]. Thus, when individuals have a clear and firm belief from which they can derive strength and guidance, they may find positive meaning in stressful events [59]. Tedeschi and Calhoun, as well, maintain that social, religious, and cultural narratives significantly affect those dealing with harsh life events and that growth as a result of trauma is shaped by the culture in which they live [61].

Another possible explanation for the higher level of personal growth reported by Arab women lies in the claim that individuals with limited resources tend to experience greater growth [62]. Those with greater personal resources are likely to cope more effectively with stressful events. Consequently, such events do not undermine or require a reevaluation of their basic values and perceptions, which is a trigger for growth [63]. Thus, higher perceived stigma and less social support may ultimately have led to more personal growth among the Arab women.

On the other hand, no difference was found here between the Arab and Jewish participants with respect to the level of stress. This is consistent with a study of female victims of domestic violence, which found that Arab and Jewish participants reported similar levels of mental distress [64]. Both groups also reported similar levels of life satisfaction. It may be assumed that for all women, the grueling process of fertility treatment is a highly private and intense experience involving considerable physical and emotional hardship. It may therefore be manifested, inter alia, in a sense of dissatisfaction with life, irrespective of ethnic affiliation [65,66]. Moreover, the fact that no difference was found between the groups in life satisfaction, whereas differences were found in personal growth, demonstrates that they are two distinct constructs.

It is important to note that the associations between stress and both personal growth and life satisfaction were not moderated by ethnicity. In other words, notwithstanding the differences between Arab and Jewish women in the level of their reported perceptions, both their reported level of stress and the associations of stress with the dependent variables were similar.

### 4.2. The Role of Stress in Life Satisfaction and Personal Growth

As predicted by Hypothesis 1, a significant negative linear relationship between level of stress and life satisfaction was found in the sample as a whole and was not moderated by ethnicity. This general finding is consistent with previous studies [25,67] suggesting that difficulty conceiving interrupts the woman’s normative life course and frustrates her expectation of motherhood, thereby diminishing her quality of life [65,66] and life satisfaction [67].

In confirmation of Hypothesis 2, a curvilinear relationship was found between stress and growth, indicating that the greatest growth was associated with moderate levels of stress. This finding, reflecting the complexity of this relationship, is in line with the results of previous studies [21] and supports the notion that extreme levels of stress can inhibit growth. Whereas very low levels may not be enough to stimulate growth, very high levels may require significant cognitive and emotional investment to process the event, not enabling the degree of emotional availability needed to grow [21,68,69]. This finding is important as it adds to the recent theoretical discussion of the role of stress in personal growth [5,40]. Furthermore, it highlights the distinct role played by stress in advancing different positive outcomes, such as life satisfaction or well-being vs. personal growth [5].

### 4.3. The Contribution of Coping Flexibility, Perceived Stigma, and Cultural Orientation to the Relationship between Stress and Growth

As predicted (Hypothesis 3), coping flexibility was found to directly contribute to personal growth, in line with previous research [28,70]. This finding reinforces the contention that coping flexibility constitutes an adaptative resource in a situation of threat or ongoing stress [71]. Moreover, a study examining the psychological state of couples dealing with fertility problems found that emotional regulation processes, such as psychological flexibility through acceptance, were a significant predictor of depressive symptoms and psychological adjustment to infertility [72]. The current study is the first, however, to examine the role of coping flexibility in the specific context of fertility treatment, as well as its relationship with personal growth. The results indicate the importance of this resource for enabling growth among women in this stressful situation.

Furthermore, coping flexibility was found to play a moderating role in the relationship between stress and personal growth, showing that only among women with lower coping flexibility was greater stress associated with less growth. It is possible that women dealing with infertility who demonstrate little coping flexibility repeatedly employ the same coping strategy, which may not always be suitable for the situation [71]. Such women may find it difficult to embrace other, more effective, alternatives that would enable them to regulate more effectively the experienced stress, and may not be emotionally available for in-depth introspection and the processing of their experience, a necessary prerequisite for the development of growth [69].

The cultural orientation of collectivism was also found to directly contribute to growth among all participants (Research question 2). People oriented toward the values of the collective tend to see themselves and their life goals as integral to their family and community, and therefore involve themselves in social life and rely on it at times of hardship [33]. The literature indicates that the tendency toward collectivism constitutes an important resource that contributes to adaptive coping by increasing the individual’s sense of support and belonging [73]. In difficult times, this positive feedback helps people to organize their internal world and perform emotional processing. Furthermore, a positive relationship has been found between social involvement, emotional availability [74], positive disclosure [75], and posttraumatic growth, suggesting that the experience of growth leans on connection with others and is even fostered when the individual feels relieved and helped after sharing their experiences [75].

In addition, both collectivism and individualism were found to moderate the relationship between stress and personal growth, but in different directions. Thus, only among women with a low sense of collectivism was greater stress associated with a lower level of growth, and only among women reporting high levels of individualism was there a negative relationship between stress and personal growth. The sense of failure and inferiority that often accompany the need for fertility treatment is heightened by social norms that threaten the woman’s sense of identity and femininity and may lead to a sense of alienation and isolation from society [2].

This reality may lead to constant tension between the desire to maintain privacy and the desire to share in order to receive support and comfort from the social group [76]. Inevitably, some women choose to keep their distance from their community and therefore do not receive its support [77]. The greater the gap between the social support they need and what they actually receive, the higher their level of stress [78,79], a situation that not only inhibits their growth, but also may ultimately affect the success of the fertility treatment itself [80].

No direct relationship was found here between perceived stigma and personal growth. This corresponds with the trend in the research that views growth as a product of a combination of factors, including internal resources and environmental variables [4,20]. Examination of the moderating role of stigma revealed that only among women reporting high levels of perceived stigma was there a negative relationship between stress and personal growth. This is another indication of the cultural dynamic involved in growth. In Israeli cultural discourse, both Arab and Jewish, female identity is defined, first and foremost, in terms of fertility and reproduction. As a result, infertile women may be stigmatized as flawed, inferior, or those who were “left behind” [81]. This increases the distress of these women, isolating them from social interactions, sharpening their feelings of guilt, worthlessness, and failure, and potentially exhausting their resources [34,38,82], again preventing them from experiencing growth.

Taken together, the findings concerning the moderating role of the cultural aspects may indicate that more vulnerable and sensitive contexts, such as a perceived social atmosphere of stigma and lower regard for communal needs, as reflected in a lower assessment of collectivism and a higher sense of individualism, heighten stress to a degree that makes it difficult to experience personal growth. In other words, there may be some circumstances in which stress and growth are unrelated and others in which they are inversely related. Considering this option against the background of the curvilinear relationship found between stress and personal growth suggests a complicated association whereby there may be different “segments” of relations between stress and growth: women who experienced a moderate level of stress during their fertility treatments reported the greatest personal growth; very low levels of stress were unrelated to growth and became more relevant as the level of stress rose to moderate levels; and higher levels of stress were associated with lower personal growth. This study therefore provides hints of circumstances or contexts that may trigger, or alternatively hinder, the experience of growth among women undergoing fertility treatment. As this is only initial evidence, more research is needed to validate this suggestion and explore additional conditions that may enable personal growth.

### 4.4. The Role of Coping Flexibility, Perceived Stigma, and Cultural Orientation in the Relationship between Stress and Life Satisfaction

In further confirmation of Hypothesis 3, a positive relationship was found between coping flexibility and life satisfaction. This is consistent with the results of a study conducted among people being treated for inflammatory bowel disease, where coping flexibility was found to enable patients to adjust more quickly to their new reality, and therefore report higher levels of life satisfaction, than those with little flexibility [83].

In contrast, no direct relationship between perceived stigma and life satisfaction was found. This runs counter to the findings of a previous study reporting a negative relationship between perceived stigma and quality of life among HIV patients [84]. However, since, to the best of our knowledge, the current study is the first to examine this relationship in women undergoing fertility treatment, the discrepancy may stem from differences in the study populations.

A positive relationship was also found between collectivism and life satisfaction. People with a sense of community belonging who perceive their support system to be available feel comfortable relying on it in a crisis. Not only does it provide them with practical aid and assistance in problem-solving, but it also serves as a reminder of their strength in difficult times. Such support has been found to contribute to psychological health and increase the individual’s life satisfaction [26]. Thus, in accordance with Lazarus and Folkman’s [3] claim that successful coping depends on the resources available to the individual, coping flexibility and collectivism were found to represent such resources in the current study.

However, none of the three factors was found to play a moderating role in the relationship between stress and life satisfaction. This suggests that the relationship between these variables is affected more by external, objective, and quantitative factors [85] and less by internal resources, such as coping flexibility, perceived stigma, and a collectivist orientation.

### 4.5. Contribution of Sociodemographic Variables to Growth and Life Satisfaction

Several background variables were found to contribute to the two outcomes. First, the more treatment cycles the participants had undergone, the lower the sense of life satisfaction they reported. This is in line with the extensive literature pointing to the negative implications of fertility treatment, which is often perceived as traumatic [86]. The lengthy and difficult process is likely to overload a woman’s resources and detract from her physical and emotional well-being, thereby reducing her satisfaction in life [13,15]. It is reasonable to assume that the longer the process, the greater its negative effects.

In addition, higher economic status was associated with both higher growth and higher life satisfaction. This is probably due to the fact that fertility treatment represents a heavy financial burden stemming from expensive medical tests and medications, lost workdays, and other costs that are not subsidized [13]. Women who are better able to cope with this drain on their resources can be expected to suffer less of a decline in life satisfaction and be more emotionally available to experience growth.

## 5. Limitations

Certain limitations of the study should be noted. First, it relies on self-report questionnaires, a subjective measure that may lead to a social desirability bias. Secondly, the data were collected at a single point in time, which did not allow for an examination of changes over time. Future longitudinal studies might reveal the trajectory of growth and life satisfaction over the course of fertility treatment. Thirdly, convenience sampling does not allow us to collect extended medical data on participants. Future studies may combine data from women with information collected from medical records to allow a fuller picture of the physical condition of women in fertility treatments. Finally, the study employed a quantitative methodology, which provides a broad picture but does not enable us to draw causal inferences. Qualitative studies using in-depth interviews might enable a deeper understanding of the multidimensional phenomena of personal growth and life satisfaction among women in fertility treatment. Future studies might also engage the women’s spouses to reveal their point of view in this challenging process.

## 6. Conclusions

The current study is, to the best of our knowledge, the first to examine the personal growth and life satisfaction of Arab and Jewish women undergoing fertility treatment. The differences found in the pattern of the independent variables associated with the two outcomes support the claim in the literature that they are distinct constructs. Whereas personal growth is the result of an internal process involving introspection and the ability to look to the future, life satisfaction is associated with current feelings and perceptions.

The major contribution of this study lies in the differences found between Arab and Jewish participants in personal growth, as well as the added value in the findings that Arab women report on higher coping flexibility and individualism. The findings further our understanding of the relationship between stress, personal growth, and life satisfaction. These findings also promote the development of a unique body of theoretical and empirical research that explores the role of culture in coping with fertility problems. This knowledge can be used to design culturally sensitive interventions for different populations of women undergoing the same stressful experience, through the understanding of the available resources which each culture provides its members with.

## Figures and Tables

**Table 1:** Distribution of Study Participants on Categorical Background Variables, by Group.

		Jewish (*n* = 100)		Arab (*n* = 105)		df	χ^2^
Variable	Category	Frequency	%	Frequency	%		
Religiosity	Religious	4	4.0	27	25.7	2	105.65 ***
	Traditional	23	23.0	74	70.5		
	Secular	73	73.0	4	3.8		
Place of birth	Abroad	16	16.0	0	0.0	1	15.72 ***
	Israel	84	84.0	90	100.0		
Employed outside the home	No	17	17.0	57	55.3	1	32.20 ***
	Yes	83	83.0	46	44.7		
Location of treatment	Private clinic	47	48.0	47	46.1	1	0.07
	Public clinic	51	52.0	55	53.9		
Children	No	71	71.0	87	83.7	1	4.67 *
	Yes	29	29.0	17	16.3		
Source of problem	Woman	35	37.2	22	21.4	3	14.57 **
	Spouse	12	12.8	32	31.1		
	Both	18	19.1	11	10.7		
	Unknown	29	30.9	38	36.9		

* *p* < 0.05, ** *p* < 0.01, *** *p* < 0.001.

**Table 2:** Means, Standard Deviations, and t Values of Continuous Background Variables, by Group.

Background Variables	Jewish (*n* = 100)			Arab (*n* = 105)			df	t
	M	SD	N	M	SD	N		
Age	34.34	5.60	96	27.51	4.78	97	185.80	9.11 ***
Economic status	2.62	0.82	99	2.57	0.83	105	202	0.79
Education	3.62	0.78	100	3.59	0.66	103	201	0.27
Years of marriage	4.90	3.56	84	5.13	3.96	105	187	0.41
Age of spouse	35.33	5.29	83	32.96	6.62	97	178	2.62 **
Months of fertility treatments	20.58	24.56	96	30.04	31.31	96	179.80	2.33 *
Number of treatment cycles	4.63	3.81	91	4.93	4.47	87	169.94	0.49

* *p* < 0.05, ** *p* < 0.01, *** *p* < 0.001.

**Table 3:** Means, Standard Deviations, and F Values of Study Variables, by Group.

	Jewish		Arab		F(1, 203)	η_p_^2^
	M	S.D.	M	S.D.		
Stress	2.54	0.49	2.47	0.45	1.16	0.006
Life satisfaction	4.18	1.25	4.33	1.23	0.80	0.004
Personal growth	3.83	0.99	4.39	0.60	24.69 ***	0.108
Perceived stigma	1.92	0.59	2.22	0.70	10.71 **	0.050
Individualism	3.48	0.37	3.62	0.39	6.20 *	0.030
Collectivism	3.68	0.33	3.70	0.33	0.10	0.000
Coping flexibility	3.00	0.38	3.41	0.37	60.89 ***	0.231

* *p* < 0.05, ** *p* < 0.01, *** *p* < 0.001.

**Table 4:** Pearson Correlations between Study Variables, by Group.

	Stress	Life Satisfaction	Personal Growth	Perceived Stigma	Individualism	Collectivism	Coping Flexibility
Stress	-	−0.42 ***	−0.24 *	0.40***	0.16	−0.18	−0.37 ***
Life satisfaction	−0.57 ***	-	0.33 **	−0.22*	0.07	0.22 *	0.23 *
Personal growth	−0.35 ***	0.59 ***	-	−0.09	0.20 *	0.36 ***	0.43 ***
Perceived stigma	0.17	−0.19	0.03	-	0.21 *	0.08	−0.01
Individualism	−0.02	0.19	0.09	0.05	-	0.20 *	0.22 *
Collectivism	0.07	0.25 *	0.22 *	0.04	0.31 **	-	0.25 *
Coping flexibility	−0.21*	0.31 **	0.35 ***	−0.004	0.26 **	0.17	-

* *p* < 0.05, ** *p* < 0.01, *** *p* < 0.001. Note: Above the diagonal for Jewish participants (*n* = 100) and below the diagonal for Arab Participants (*n* = 105).

**Table 5:** Results of the Regression Analyses for Personal Growth and Life Satisfaction.

	Personal Growth				Life Satisfaction	
	β	t	∆R^2^	β	t	∆R^2^
**Step 1**			0.15 ***			0.217 ***
Ethnicity ^a^	0.32	3.93 ***		0.06	0.81	
Age	−0.06	−0.75		−0.08	−1.03	
Economic status	0.14	2.18 *		0.38	6.06 ***	
Months of fertility treatments	−0.08	−0.93		0.05	0.68	
Fertility treatment cycles	−0.07	−0.86		−0.24	−3.13 **	
Children ^b^	−0.004	0.05		0.15	2.23	
R^2^	0.15 ***			0.22 ***		
F	5.81 ***			9.14 ***		
**Step 2**			0.054 ***			0.188 ***
Stress	−0.24	−3.66 **		−0.45	−7.89 ***	
R^2^	0.204 ***			0.41 ***		
F	7.20 ***			19.14 ***		
**Step 3**			0.119 ***			0.056 **
Coping flexibility	0.31	4.06 ***		0.14	2.06 *	
Perceived stigma	0.01	0.17		−0.08	−1.31	
Individualism	0.01	0.21		0.07	1.26	
Collectivism	0.17	2.68 **		0.14	2.46 *	
R^2^	0.323 ***			0.46 ***		
F	8.37 ***			14.99 ***		
**Step 4**			0.08 ***			0.011
Stress X Stigma	−0.12	−1.99 *		−0.10	−1.82	
Stress X Coping flexibility	0.18	2.83 **		−0.04	−0.67	
Stress X Individualism	−0.14	−2.15 *		−0.04	−0.65	
Stress X Collectivism	0.19	3.13 **		−0.02	−0.36	
Stress X Ethnicity	−0.03	−0.26		−0.16	−1.69	
R^2^	0.403 ***			0.472 ***		
F	8.45 ***			11.26 ***		
R^2^	0.403 ***			0.472 ***		
F	6.69			8.70 ***		

* *p* < 0.05, ** *p* < 0.01, *** *p* < 0.001; ^a^ 0 = Arab, 1 = Jewish; ^b^ 0 = No, 1 = Yes.

**Table 6:** Results of the Regression Testing for a Curvilinear Relationship between Stress and Personal Growth.

	β	t	∆R^2^
**Step 1**			0.150
Ethnicity ^a^	0.32	3.93 ***	
Age	−0.06	−0.75	
Economic status	0.14	2.18 *	
Months of fertility treatments	−0.08	−0.93	
Fertility treatment cycles	−0.07	−0.86	
Children ^b^	−0.004	−0.05	
**Step 2**			0.054 ***
Stress	−0.24	−3.66 ***	
**Step 3**			0.073 **
Stress^2^	−0.27	−4.40 ***	
R^2^			0.277
F(8, 196)			9.37 ***

* *p* < 0.05, ** *p* < 0.01; *** *p* < 0.001; ^a^ 0 = Arab, 1 = Jewish; ^b^ 0 = No, 1 = Yes.

## Data Availability

The datasets generated during the current study are available from the corresponding author on reasonable request.

## References

[1] Cousineau T.M., Domar A.D. (2007). Psychological impact of infertility. Best Pract. Res. Clin. Obstet. Gynecol..

[2] Daibes M.A., Safadi R.R., Athamneh T., Anees I.F., Constantino R.E. (2018). ‘Half a woman, half a man; that is how they make me feel’: A qualitative study of rural Jordanian women’s experience of infertility. Cult. Health Sex..

[3] Lazarus R.S., Folkman S. Stress, Appraisal, and Coping.

[4] Schaefer J.A., Moos R.H., Tedeschi R.G., Park C., Calhoun L.G. (1998). The context for posttraumatic growth: Life crises, individual and social resources, and coping. Posttraumatic Growth: Positive Changes in the Aftermath of Crisis.

[5] Tedeschi R.G., Shakespeare-Finch J., Taku K., Calhoun L.G. (2018). Posttraumatic Growth. Theory, Research, and Applications.

[6] Kong L., Fang M., Ma T., Li G., Yang F., Meng Q., Li Y., Li P. (2018). Positive affect mediates the relationships between resilience, social support and posttraumatic growth of women with infertility. Psychol. Health Med..

[7] Yu Y., Peng L., Chen L., Long L., He W., Li M., Wang T. (2014). Resilience and social support promote posttraumatic growth of women with infertility: The mediating role of positive coping. Psychiatry Res..

[8] Zegers-Hochschild F., Adamson G.D., de Mouzon J., Ishihara O., Mansour R., Nygren K., Sullivan E., van der Poel S. (2009). The International Committee for Monitoring Assisted Reproductive Technology (ICMART) and the World Health Organization (WHO) revised glossary on ART terminology, 2009. Hum. Reprod..

[9] (2015). Ministry of Health. http://www.health.gov.il/PublicationsFiles/IVF1986_2012.pdf.

[10] Birenbaum-Carmeli D. (2016). Thirty-five years of assisted reproductive technologies in Israel. Reprod. Biomed. Soc. Online.

[11] Chandra A., Copen C.E., Stephen E.H. (2014). Infertility Service Use in the United States: Data from the National Survey of Family Growth, 1982–2010 (No. 1250).

[12] Grant L.E., Cochrane S. (2014). Acupuncture for the mental and emotional health of women undergoing IVF treatment: A comprehensive review. Aust. J. Acupunct. Chin. Med..

[13] Benyamini Y., Gefen-Bardarian Y., Gozlan M., Tabiv G., Shiloh S., Kokia E. (2008). Coping specificity: The case of women coping with infertility treatments. Psychol. Health.

[14] Madge V. (2011). Infertility, women and assisted reproductive technologies: An exploratory study in Pune, India. Indian J. Gend. Stud..

[15] Papadatou D., Papaligoura Z.G., Bellali T. (2016). From infertility to successful third-party reproduction: The trajectory of Greek women. Qual. Health Res..

[16] Culley L., Hudson N., Van Rooij F. (2012). Marginalized Reproduction: Ethnicity, Infertility and Assisted Reproductive Technologies.

[17] Patel A., Sharma P.S.V.N., Narayan P., Binu V.S., Dinesh N., Pai P.J. (2016). Prevalence and predictors of infertility-specific stress in women diagnosed with primary infertility: A clinic-based study. J. Hum. Reprod. Sci..

[18] Paul M.S., Berger R., Berlow N., Rovner-Ferguson H., Figlerski L., Gardner S., Malave A.F. (2010). Posttraumatic growth and social support in individuals with infertility. Hum. Reprod..

[19] Valsangkar S., Bodhare T., Bele S., Sai S. (2011). An evaluation of the effect of infertility on marital, sexual satisfaction indices and health-related quality of life in women. J. Hum. Reprod. Sci..

[20] Tedeschi R.G., Calhoun L.G. (2004). Posttraumatic growth: Conceptual foundation and empirical evidence. Psychol. Inq..

[21] Rozen G., Taubman-Ben-Ari O., Strauss T., Morag I. (2018). Personal growth of mothers of preterms: Objective severity of the event, subjective stress, personal resources, and maternal emotional support. J. Happiness Stud..

[22] Berger R., Paul M.S., Henshaw L.A. (2013). Women’s experience of infertility: A multi-systemic perspective. J. Int. Women Stud..

[23] Diener E. (1984). Subjective well-being. Psychol. Bull..

[24] Huebner E.S., Gilman R., Laughlin J.E. (1999). A multimethod investigation of the multidimensionality of children’s well-being reports: Discriminant validity of life satisfaction and self-esteem. Soc. Indic. Res..

[25] Maroufizadeh S., Ghaheri A., Samani R.O., Ezabadi Z. (2016). Psychometric properties of the Satisfaction with Life Scale (SWLS) in Iranian infertile women. Int. J. Reprod. Biomed..

[26] Dembińska A.A. (2016). Psychological determinants of life satisfaction in women undergoing infertility treatment. Health Psychol. Rep..

[27] McCracken L.M., Morley S. (2014). The psychological flexibility model: A basis for integration and progress in psychological approaches to chronic pain management. J. Pain.

[28] Cohen O., Katz M. (2015). Grief and growth of bereaved siblings as related to attachment style and flexibility. Death Stud..

[29] Verhaak C.M., Smeenk J.M.J., Nahuis M.J., Kremer J.A.M., Braat D.D.M. (2007). Long-term psychological adjustment to IVF/ICSI treatment in women. Hum. Reprod..

[30] Greil A.L., Slauson-Blevins K., McQuillan J. (2010). The experience of infertility: A review of recent literature. Sociol. Health Illn..

[31] Pines A.M., Zaidman N. (2003). Gender, culture, and social support: A male–female, Israeli Jewish-Arab comparison. Sex Roles.

[32] Kasler J., Zysberg L., Gal R. (2021). Culture, collectivism-individualism and college student plagiarism. Ethics Behav..

[33] Triandis H.C. (1995). Individualism and Collectivism.

[34] Inhorn M.C. (2006). Making Muslim babies: IVF and gamete donation in Sunni versus Shi’a Islam. Cult. Med. Psychiatry.

[35] Obeidat H.M., Hamlan A.M., Callister L.C. (2014). Missing motherhood: Jordanian women’s experiences with infertility. Adv. Psychiatry.

[36] Brehm S.S., Kassin S.M., Fein S. (1999). Social identity theory. Social Psychology.

[37] Missmer S.A., Seifer D.B., Jain T. (2011). Cultural factors contributing to health care disparities among patients with infertility in Midwestern United States. Fertil. Steril..

[38] Serour G.I. (2008). Islamic perspectives in human reproduction. Reprod. Biomed. Online.

[39] Moura-Ramos M., Gameiro S., Canavarro M.C., Soares I. (2012). Assessing infertility stress: Re-examining the factor structure of the Fertility Problem Inventory. Hum. Reprod..

[40] Taubman-Ben-Ari O., Taubman-Ben-Ari O. (2019). Blossoming and growing in the transition to parenthood. Pathways and Barriers to the Transition to Parenthood—Existential Concerns Regarding Fertility, Pregnancy, and Early Parenthood.

[41] Kiesswetter M., Marsoner H., Luehwink A., Fistarol M., Mahlknecht A., Duschek S. (2020). Impairments in life satisfaction in infertility: Associations with perceived stress, affectivity, partnership quality, social support and the desire to have a child. Behav. Med..

[42] Cohen S., Kamarck T., Mermelstein R. (1983). A global measure of perceived stress. J. Health Soc. Behav..

[43] Tedeschi R.G., Calhoun L.G. (1996). The Posttraumatic Growth Inventory: Measuring the positive legacy of trauma. J. Trauma. Stress.

[44] Diener E.D., Emmons R.A., Larsen R.J., Griffin S. (1985). The Satisfaction with Life Scale. J. Personal. Assess..

[45] Vriezekolk J.E., van Lankveld W.G., Eijsbouts A.M., van Helmond T., Geenen R., van den Ende C.H. (2012). The Coping Flexibility Questionnaire: Development and initial validation in patients with chronic rheumatic diseases. Rheumatol. Int..

[46] Singelis T.M., Triandis H.C., Bhawuk D.P., Gelfand M.J. (1995). Horizontal and vertical dimensions of individualism and collectivism: A theoretical and measurement refinement. Cross Cult. Res..

[47] Komiya N., Good G.E., Sherrod N.B. (2000). Emotional openness as a predictor of college students’ attitudes toward seeking psychological help. J. Couns. Psychol..

[48] Naab F., Brown R., Heidrich S. (2013). Psychosocial health of infertile Ghanaian women and their infertility beliefs. J. Nurs. Scholarsh..

[49] Hayes A.F. (2013). Methodology in the Social Sciences. Introduction to Mediation, Moderation, and Conditional Process Analysis: A Regression-based Approach.

[50] Bar H. (2017). Missing data—Mechanisms and possible solutions. Cult. Educ..

[51] Little R.J. (1988). A test of missing completely at random for multivariate data with missing values. J. Am. Stat. Assoc..

[52] Cohen A. (2006). The relationship between multiple commitments and organizational citizenship behavior in Arab and Jewish culture. J. Vocat. Behav..

[53] Dwairy M.A. (2006). Counseling and Psychotherapy with Arabs and Muslims: A Culturally Sensitive Approach.

[54] Joseph S. (1999). Intimate Selving in Arab Families: Gender, Self, and Identity.

[55] Rashad H., Osman M.I., Roudi-Fahimi F. (2005). Marriage in the Arab World.

[56] Hammoudeh D., Hamayel L., Abu-Rmeileh N.M., Giacaman R. (2013). Effect of infertility on women in the occupied Palestinian territory: A pilot qualitative study. Lancet.

[57] Dwairy M. (2009). Culture-analysis and metaphor therapy with Arab-Muslim clients. J. Clin. Psychol..

[58] Fido A., Zahid M.A. (2004). Coping with infertility among Kuwaiti women: Cultural perspectives. Int. J. Soc. Psychiatry.

[59] Ghafouri S.F., Ghanbari S., Fallahzadeh H., Shokri O. (2016). The relation between marital adjustment and posttraumatic growth in infertile couples: The mediatory role of religious coping strategies. J. Reprod. Infertil..

[60] Ng G.C., Mohamed S., Sulaiman A.H., Zainal N.Z. (2017). Anxiety and depression in cancer patients: The association with religiosity and religious coping. J. Relig. Health.

[61] Calhoun L.G., Cann A., Tedeschi R.G., Weiss T., Berger R. (2010). The posttraumatic growth model: Sociocultural considerations. Posttraumatic Growth and Culturally Competent Practice: Lessons Learned from around the Globe.

[62] Taubman-Ben-Ari O., Shaver P.R., Mikulincer M. (2012). Becoming and developing: Personal growth in the wake of parenthood and grandparenthood. Meaning, Mortality, and Choice—The Social Psychology of Existential Concerns.

[63] Park C.L., Fenster J.R. (2004). Stress-related growth: Predictors of occurrence and correlates with psychological adjustment. J. Soc. Clin. Psychol..

[64] Dekel R., Shaked O.Z., Ben-Porat A., Itzhaky H. (2019). Posttraumatic stress disorder upon admission to shelters among female victims of domestic violence: An ecological model of trauma. Violence Vict..

[65] Benyamini Y., Gozlan M., Weissman A. (2017). Normalization as a strategy for maintaining quality of life while coping with infertility in a pronatalist culture. Int. J. Behav. Med..

[66] Rooney K.L., Domar A.D. (2016). The impact of stress on fertility treatment. Curr. Opin. Obstet. Gynecol..

[67] McQuillan J., Torres Stone R.A., Greil A.L. (2007). Infertility and life satisfaction among women. J. Fam. Issues.

[68] Taku K., Cann A., Tedeschi R.G., Calhoun L.G. (2015). Core beliefs shaken by an earthquake correlate with posttraumatic growth. Psychol. Trauma Theory Res. Pract. Policy.

[69] Triplett K.N., Tedeschi R.G., Cann A., Calhoun L.G., Reeve C.L. (2012). Posttraumatic growth, meaning in life, and life satisfaction in response to trauma. Psychol. Trauma Theory Res. Pract. Policy.

[70] Buser J.K., Kearney A. (2017). Stress, adaptive coping, and life satisfaction. J. Coll. Couns..

[71] Cheng C., Lau H.P.B., Chan M.P.S. (2014). Coping flexibility and psychological adjustment to stressful life changes: A meta-analytic review. Psychol. Bull..

[72] Pinto-Gouveia J., Galhardo A., Cunha M., Matos M. (2012). Protective emotional regulation processes towards adjustment in infertile patients. Hum. Fertil..

[73] Williams P., Barclay L., Schmied V. (2004). Defining social support in context: A necessary step in improving research, intervention, and practice. Qual. Health Res..

[74] Prati G., Pietrantoni L. (2009). Optimism, social support, and coping strategies as factors contributing to posttraumatic growth: A meta-analysis. J. Loss Trauma.

[75] Taku K., Tedeschi R.G., Shakespeare-Finch J., Krosch D., David G., Kehl D., Grunwald S., Romeo A., Di Tella M., Kamibeppu K. (2021). Posttraumatic growth (PTG) and posttraumatic depreciation (PTD) across ten countries: Global validation of the PTG-PTD theoretical model. Personal. Individ. Differ..

[76] Steuber K.R., Solomon D.H. (2011). Factors that predict married partners’ disclosures about infertility to social network members. J. Appl. Commun. Res..

[77] Raque-Bogdan T.L., Hoffman M.A. (2015). The relationship among infertility, self-compassion, and well-being for women with primary or secondary infertility. Psychol. Women Q..

[78] Levi-Belz Y. (2015). Stress-related growth among suicide survivors: The role of interpersonal and cognitive factors. Arch. Suicide Res..

[79] Levi-Belz Y. (2016). To share or not to share? The contribution of self-disclosure to stress-related growth among suicide survivors. Death Stud..

[80] Karlidere T., Bozkurt A., Ozmenler K.N., Ozsahin A., Kucuk T., Yetkin S. (2008). The influence of emotional distress on the outcome of in-vitro fertilization (IVF) and/or intracytoplasmic sperm injection (ICSI) treatment among infertile Turkish women. Isr. J. Psychiatry Relat. Sci..

[81] Galhardo A., Cunha M., Pinto-Gouveia J., Matos M. (2013). The mediator role of emotion regulation processes on infertility-related stress. J. Clin. Psychol. Med. Settings.

[82] Agostini F., Monti F., De Pascalis L., Paterlini M., La Sala G.B., Blickstein I. (2011). Psychosocial support for infertile couples during assisted reproductive technology treatment. Fertil. Steril..

[83] Rudnik A., Piotrowicz G., Basińska M.A., Rashedi V. (2019). The importance of cognitive flexibility and flexibility in coping with stress for the quality of life in inflammatory bowel disease patients during biological therapy: A preliminary report. Przegląd Gastroenterol..

[84] Shrestha R., Altice F.L., Copenhaver M.M. (2019). HIV-related stigma, motivation to adhere to antiretroviral therapy, and medication adherence among HIV-positive methadone-maintained patients. J. Acquir. Immune Defic. Syndr..

[85] Felce D., Perry J., Renwick I., Brown I., Nagler M. (1996). Exploring current conceptions of quality of life. Quality of Life in Health Promotion and Rehabilitation: Conceptual Approaches, Issues, and Application.

[86] Benyamini Y., Gozlan M., Kokia E. (2009). Women’s and men’s perceptions of infertility and their associations with psychological adjustment: A dyadic approach. Br. J. Health Psychol..

